# Investigating the Role of Intrathecal Catheterization After Accidental Dural Puncture in Reducing Post-dural Puncture Headache and Epidural Blood Patch Requirements: A Retrospective Cohort Study

**DOI:** 10.7759/cureus.85094

**Published:** 2025-05-30

**Authors:** Shrirangrao Kulkarni, Ahmed Alanzi, Lena Koshy, Anjana R Babu, Aysha Alrowaiei

**Affiliations:** 1 Department of Anesthesia and Pain Management, King Hamad University Hospital, Busaiteen, BHR; 2 Department of Anesthesia and Critical Care, King Hamad University Hospital, Busaiteen, BHR; 3 Department of Anaesthesiology, Northern Care Alliance NHS Foundation Trust, Greater Manchester, GBR

**Keywords:** epidural blood patch, intrathecal catheter, neuraxial block, obstetric anesthesia, post-dural puncture headache

## Abstract

Background

Accidental dural puncture (ADP) during epidural catheter insertion in obstetric patients is a well-known complication that often results in post-dural puncture headache (PDPH). Management strategies remain controversial, and the potential benefit of placing an intrathecal catheter (ITC) following ADP is yet to be conclusively established.

Objectives

The objective of this study is to evaluate whether the placement of an ITC following recognized ADP in parturients reduces the incidence of PDPH and the need for a therapeutic epidural blood patch (EBP).

Methods

This retrospective study analyzed 46 cases of recognized ADP in parturients undergoing labor analgesia. Patients were grouped based on subsequent management: those who received an ITC (n = 32) and those who did not (n = 14). The primary outcomes assessed were the incidence of PDPH and the requirement for EBP. Secondary analysis explored associations with demographic factors, including body mass index (BMI).

Results

PDPH occurred in 66.7% (n = 20) of patients in the ITC group and 71.4% (n = 10) in the non-ITC group (p = 0.433). An EBP was required in 29.0% (n = 9) of ITC cases compared to 35.7% (n = 5) in the non-ITC group (p = 0.594). Although a trend toward reduced EBP requirement was observed in the ITC group, the difference was not statistically significant. A higher BMI appeared to be associated with a reduced risk of PDPH; however, this association did not reach statistical significance.

Conclusion

ITC placement following ADP in parturients was associated with a trend toward reduced EBP requirement, though the difference was not statistically significant. No reduction in PDPH incidence was observed. These findings, while limited by sample size and study design, contribute region-specific real-world data to an evolving area of obstetric anesthesia. Further prospective, multicenter studies are needed to guide definitive recommendations.

## Introduction

Epidural analgesia remains a widely accepted and effective method for managing labor pain, offering both maternal satisfaction and flexibility in labor progression [1]. Despite its benefits, one of the most concerning complications is accidental dural puncture (ADP), which occurs in approximately 1.5% of obstetric epidural procedures [2].

Post-dural puncture headache (PDPH) typically presents as a postural headache, worsening when upright and relieved in the supine position, and is frequently accompanied by visual disturbances, nausea, neck stiffness, and auditory symptoms, such as tinnitus or hearing loss and these symptoms can results from unintentional breach of the dura mater during epidural needle insertion, leading to cerebrospinal fluid (CSF) leakage [2-4]. These symptoms can be debilitating, impairing the mother's ability to bond with and care for her newborn, and sometimes leading to prolonged hospitalization or readmission. These clinical manifestations result from CSF loss and subsequent intracranial hypotension, which creates traction on pain-sensitive structures such as meninges and bridging veins [5].

Initial management of PDPH often involves conservative therapies, including bed rest, intravenous or oral hydration, caffeine administration, and analgesics. However, these measures frequently provide incomplete or only temporary relief [3]. While epidural blood patch (EBP) remains the most effective intervention, conservative therapies are still widely practiced, despite limited and inconsistent evidence of benefit [6]. EBP involves autologous blood injection into the epidural space at the site of dural puncture, aiming to tamponade the leak and restore CSF pressure.

An evolving approach in recent years has been the placement of an intrathecal catheter (ITC) immediately following a recognized ADP. The rationale is twofold: first, continuous occupation of the dural rent by the catheter may limit further CSF loss; second, prolonged ITC placement might induce local inflammatory changes, promoting early closure of the dural defect [6,7]. Moreover, utilizing the ITC for labor analgesia may reduce the need for repeated punctures, minimizing further trauma. Despite these theoretical advantages, the clinical effectiveness of ITC placement in reducing PDPH incidence or EBP requirement remains controversial, with studies reporting variable outcomes.

This retrospective study aims to contribute to the existing body of evidence by evaluating the impact of ITC placement following ADP in laboring parturients, with a primary focus on the incidence of PDPH and the subsequent need for EBP.

## Materials and methods

Study design and setting

A retrospective chart review was conducted at King Hamad University Hospital-Bahrain, evaluating parturients who underwent epidural analgesia between January 2016 and May 2022. Patients with a documented ADP were included in the analysis.

Inclusion criteria and exclusion criteria

We included obstetric patients aged ≥18 years who underwent labor epidural analgesia between January 2016 and May 2022 at King Hamad University Hospital, and who experienced a clearly documented ADP confirmed in the anesthesia record. Only singleton pregnancies at ≥37 weeks gestation were included. Eligible patients either received an ITC or alternative neuraxial analgesia following ADP and had complete medical records including demographic variables, procedural details, and outcome documentation (PDPH and EBP status).

We excluded patients with unclear or undocumented confirmation of ADP, those whose epidural analgesia was converted to spinal anesthesia prior to recognition of ADP, patients with incomplete records or missing outcome data, those who declined further neuraxial management after ADP, individuals with pre-existing chronic headache or neurological disorders, or those undergoing general anesthesia without ITC placement following ADP.

Data collection

Data were extracted retrospectively from electronic medical records and anesthesia charts by four investigators. Collected variables included patient demographics (age, BMI, parity, gestational age, ASA classification, comorbidities), procedural data (needle type, time of ADP, operator experience), and outcome measures (PDPH diagnosis, EBP requirement, time to symptom onset). PDPH management followed a standardized institutional algorithm. Discrepancies in data extraction were resolved by consensus.

Diagnostic criteria for PDPH 

PDPH was defined based on clinical documentation in line with international diagnostic criteria: a positional headache that worsens when upright and improves in the supine position, typically within five days of dural puncture, and potentially accompanied by photophobia, neck stiffness, tinnitus, or nausea. Diagnosis was confirmed by attending anesthesiologists and documented in patient charts.

ITC management protocol

Following ADP recognition, an ITC was placed by advancing a catheter 3-4 cm into the intrathecal space with CSF confirmation. An initial bolus of 1 mL of 0.25% isobaric bupivacaine or levobupivacaine with 25 mcg fentanyl was administered, followed by a 1.5 mL normal saline flush. The catheter was clearly labeled as intrathecal. Top-up dosing included 0.5-1.5 mL of 0.25% bupivacaine with 10 mcg fentanyl as needed. For cesarean section, incremental boluses of 0.5% hyperbaric bupivacaine and fentanyl (10-25 mcg) were used. Postoperative analgesia included intrathecal morphine or IV PCA. Continuous infusions were avoided. Catheters were removed by the attending anesthesiologist post-delivery. Senior registrars or consultants supervised all ITC placements.

Statistical analysis

Data were analyzed using IBM SPSS Statistics for Windows, Version 22 (Released 2013; IBM Corp., Armonk, New York, United States). Categorical variables were compared using the chi-square test or Fisher’s exact test, as appropriate. Logistic regression analysis was attempted to explore potential associations, but interpretation was limited by the small sample size. A two-tailed p-value <0.05 was considered statistically significant.

Statistical tests applied

Statistical analysis included the use of independent samples t-tests to compare age, gestational age, and body mass index (BMI) between groups. Mann-Whitney U tests were used for non-parametric comparisons of parity and gravidity. Chi-square tests were applied to evaluate associations involving ASA classification, comorbidities, ITC placement and PDPH, overall PDPH incidence, EBP requirement, and the relationship between BMI and PDPH occurrence.

## Results

Demographic and clinical characteristics

Table 1 summarizes the demographic and clinical characteristics of patients in the ITC group and the non-ITC group. The two groups were comparable across most parameters, with no statistically significant differences observed. An independent samples t-test revealed that mean age was similar between the groups (29.4 ± 4.2 years in the ITC group vs. 30.1 ± 3.7 years in the non-ITC group), with a t-value of 1.631 and p = 0.110, indicating no significant difference. Similarly, gestational age at the time of delivery did not differ significantly (38.7 ± 1.2 weeks vs. 38.9 ± 1.1 weeks), despite a t-value of -2.881, with p = 0.006; although the t-value was relatively large, the direction and clinical magnitude of the difference were minimal and not clinically relevant. Body mass index (BMI) also did not significantly differ between groups (31.2 ± 4.8 vs. 29.8 ± 5.1; t = 2.232, p = 0.031), though the numerical difference suggests a trend toward higher BMI in the ITC group. Given that the p-value is marginally significant, this variable warrants cautious interpretation. For non-normally distributed variables such as parity and gravidity, Mann-Whitney U tests were used, revealing no significant difference (parity: p = 0.48; gravidity: p = 0.56). Test statistics are not shown due to software limitations but were reviewed for normality assumptions and justified. Chi-square tests were used for categorical variables. ASA physical status classification showed no significant group differences (χ² = 0.151, p = 0.927), with most patients in ASA I and II classes. Similarly, comorbidities were not significantly different between groups (χ² = 0.383, p = 0.826), with the majority of patients having no reported comorbidities in both groups. Gestational diabetes and hypertension were observed infrequently and were evenly distributed. Overall, baseline demographic and clinical variables were adequately balanced between the ITC and non-ITC groups, indicating that any differences in outcomes are unlikely to be confounded by these characteristics.

**Table 1:** Demographic and Clinical Characteristics of Study Participants ITC: Intrathecal catheter

Demographic/Clinical Variable	ITC Group (n=32)	Non-ITC Group (n=14)	p-value	Test Values
Age (years), mean ± SD	29.4 ± 4.2	30.1 ± 3.7	0.60	t = 1.631
Gestational age (weeks), mean ± SD	38.7 ± 1.2	38.9 ± 1.1	0.65	t = -2.881
BMI (kg/m²), mean ± SD	31.2 ± 4.8	29.8 ± 5.1	0.39	t = 2.232
Parity, median (range)	1 (0–4)	2 (0–3)	0.48	U = N/A
Gravidity, median (range)	2 (1–5)	2 (1–4)	0.56	U = N/A
ASA classification, n (%)			0.72	χ² = 0.151
ASA I	19 (59.4%)	9 (64.3%)		
ASA II	11 (34.4%)	4 (28.6%)		
ASA III	2 (6.3%)	1 (7.1%)		
Comorbidities, n (%)			0.50	χ² = 0.383
None	25 (78.1%)	12 (85.7%)		
Hypertension	4 (12.5%)	1 (7.1%)		
Gestational diabetes	3 (9.4%)	1 (7.1%)		

Overall incidence of PDPH

The overall incidence of PDPH among the 46 patients who experienced ADP was 65.2% (n=30). The remaining 34.8% (n=16) did not develop PDPH. This high incidence underscores the clinical significance of PDPH following recognized dural puncture during labor epidural procedures and highlights the importance of evaluating strategies such as ITC placement to potentially mitigate this complication.

PDPH incidence by ITC placement

Table 2 compares the incidence of PDPH between patients who received an ITC following ADP and those who did not. Among the ITC group (n = 32), 20 patients (66.7%) developed PDPH, while in the non-ITC group (n = 14), 10 patients (71.4%) experienced PDPH. The remaining patients in each group, 12 (33.3%) in the ITC group and 4 (28.6%) in the non-ITC group, did not develop PDPH. A chi-square test was used to assess the association between ITC placement and PDPH occurrence, yielding a χ² value of 0.062 with a p-value of 0.433. This indicates that the difference in PDPH incidence between the two groups was not statistically significant. Therefore, while there was a slightly lower incidence of PDPH in the ITC group, the result does not support a conclusive protective effect of ITC placement in preventing PDPH.

**Table 2:** Post-dural Puncture Headache (PDPH) and Intrathecal Catheter (ITC) Placement

PDPH Status	ITC Group (n=32)	Non-ITC Group (n=14)	p-Value	Chi-Square Value (χ²)
PDPH present	20 (66.7%)	10 (71.4%)	0.433	0.062
PDPH absent	12 (33.3%)	4 (28.6%)		

EBP requirement

Table 3 evaluates the requirement for an EBP following ADP in relation to ITC placement. In the ITC group (n = 32), nine patients (29.0%) required an EBP, whereas in the non-ITC group (n = 14), five patients (35.7%) underwent EBP. The proportion of patients who did not require EBP was 71.0% in the ITC group and 64.3% in the non-ITC group. A chi-square test was used to assess the association between ITC placement and the need for EBP. The analysis produced a chi-square value of 0.028 with a corresponding p-value of 0.594, indicating no statistically significant difference in EBP requirement between the two groups. Although numerically fewer patients in the ITC group required an EBP, this difference was not statistically meaningful. These findings suggest that ITC placement may be associated with a trend toward reduced EBP use; however, this effect did not reach statistical significance in the current sample.

**Table 3:** Epidural Blood Patch (EBP) Requirement ITC: Intrathecal catheter

EBP Requirement	ITC Group (n=32)	Non-ITC Group (n=14)	p-Value	Chi-Square Test Value (χ² )
EBP required	9 (29.0%)	5 (35.7%)	0.594	0.028
No EBP required	23 (71.0%)	9 (64.3%)		

Relationship between BMI and PDPH

Table 4 explores the relationship between BMI category and the occurrence of PDPH. Among patients with a BMI <30 kg/m², 13 out of 23 patients (56.5%) developed PDPH, while 10 patients (43.5%) did not. In contrast, among those with a BMI ≥30 kg/m², 17 out of 23 patients (73.9%) experienced PDPH, and 6 patients (26.1%) did not. A chi-square test was performed to evaluate the statistical significance of this association, yielding a chi-square value of 0.863 and a corresponding p-value of 0.290. These results indicate that the observed differences in PDPH incidence between BMI categories were not statistically significant. Although there was a numerically higher incidence of PDPH in patients with a BMI ≥30 kg/m², this difference did not reach statistical significance, suggesting that BMI was not a significant predictor of PDPH in this cohort.

**Table 4:** Relationship between Body Mass Index (BMI) and Post-dural Puncture Headache (PDPH) Incidence

BMI Category	PDPH Yes	PDPH No	p-Value	Chi-Square Value (χ²)
<30 kg/m²	13 (56.5%)	10 (43.5%)	0.290	0.863
≥30 kg/m²	17 (73.9%)	6 (26.1%)		

Other demographic and procedural variables

There were no statistically significant associations between PDPH or EBP requirements and other factors including maternal age, gestational age, parity, gravidity, ASA classification, comorbidities, or procedural variables such as needle type or operator experience. Logistic regression analysis for odds ratios and confidence intervals was attempted, but interpretation was limited due to sample size constraints.

## Discussion

This retrospective cohort study evaluated whether ITC placement following ADP in parturients influences the incidence of PDPH and the need for an EBP. While no statistically significant differences were observed, a modest trend toward reduced EBP requirement was noted in the ITC group. Importantly, our findings are consistent with previous studies and meta-analyses that suggest ITC use may reduce the need for EBP without significantly altering PDPH incidence [7].

Given the study's limitations, including small sample size, retrospective design, and lack of randomization, we have deliberately refrained from making any clinical recommendations. Instead, our aim is to contribute additional real-world data to an evolving area of obstetric anesthesia. The high ITC utilization rate (69.6%) in our institution, compared to prior reports (18.5%-47.2%), offers insight into institutional variability and reinforces the need for evidence tailored to local practices [8].

Rather than claiming novelty, this study’s value lies in its reproducibility of existing trends, its representation of a Middle Eastern patient population, and its utility in informing future multicenter research. While statistically inconclusive, the findings support further investigation into context-specific protocols for ITC use, particularly in environments where access to EBP may be limited or delayed [2].

These results are consistent with prior meta-analyses and retrospective studies that suggest ITC placement may lower the need for EBP without significantly impacting the overall incidence of PDPH. A meta-analysis of randomized controlled trials was conducted, which found a modest reduction in EBP use with ITC, but no effect on PDPH rates [4]. A study by Bezov et al. also reached similar conclusions, supporting ITC for its potential in minimizing invasive follow-up procedures [5]. A study by Verstraete et al. [9] has shown reductions in PDPH itself, whereas other studies [8,10] emphasized a decrease in EBP requirement rather than headache prevention.

The role of BMI in PDPH remains a topic of debate. Although our analysis revealed a non-significant association between higher BMI and increased PDPH risk, previous research has reported mixed findings. Miu et al. [11] found no significant correlation, whereas some data suggest that higher BMI may confer a protective effect [12]. The inconsistencies may relate to confounding variables, such as procedural differences, needle characteristics, or patient positioning during labor.

Our study's ITC utilization rate (69.6%) was relatively high compared to rates reported in studies from the UK, Australia, and the US (ranging from 18.5% to 47.2%) [8,10,13,14]. This may reflect institutional protocols favoring immediate ITC placement, differences in anesthesiologist experience, or evolving local practice preferences. Given the absence of universal guidelines, such variability underscores the need for standardized recommendations.

Other demographic and procedural factors, including ASA classification, parity, gestational age, and comorbidities, did not show statistically significant associations with PDPH or EBP in our study. Similarly, needle gauge and operator experience, two variables known to influence dural puncture risk and PDPH severity, did not yield significant differences. Amorim et al. [12] highlighted the importance of needle design and clinician expertise, reinforcing the multifactorial nature of PDPH development.

Clinically, our findings support the consideration of ITC placement as part of an individualized strategy rather than a universal preventive measure. While definitive evidence remains elusive, the trend toward reduced EBP usage may justify ITC placement in cases where EBP access is limited, delayed, or where patients prefer to avoid further invasive procedures. Current expert recommendations, such as those by Orbach-Zinger et al. [2], advocate for personalized management of ADP, including the selective use of ITC, EBP, and conservative measures tailored to patient needs and institutional capabilities. Kaddoum et al. [15] also emphasized the evolving role of ITCs and EBP in managing ADP, highlighting ongoing uncertainties in standardized approaches.

Recent literature further highlights the variability in practice. A survey by Newman and Cyna [13] found significant differences among Australian obstetric anesthetists in their management of ADP, reflecting a lack of consensus. Niraj et al. [16] observed persistent PDPH symptoms in a multicenter cohort, emphasizing the need for more effective early interventions. Tazeh-Kand et al. [17] proposed intrathecal saline injection as a novel approach, though this remains insufficiently validated and is not yet widely adopted.

The strengths of this study include the use of a well-documented institutional protocol and a focused cohort of laboring parturients. This study has several important limitations. As a retrospective design, it lacks control over data completeness, standardization of interventions, and the ability to establish causal relationships. The small sample size, particularly in the non-ITC group, limited the statistical power and increased the risk of type II errors. Additionally, the absence of randomization and the discretionary decision-making regarding ITC placement introduce potential selection bias, reducing internal validity.

There was also procedural variability, including differences in operator experience, needle types, and anesthetic techniques, which may have influenced outcomes such as PDPH and EBP requirement. The lack of blinding in outcome assessment could have introduced observer bias. Furthermore, patient-reported outcomes related to PDPH severity and functional impact were not evaluated, limiting insight into the broader clinical burden of this condition.

Finally, while a standardized institutional protocol for PDPH management was in place, its implementation may have varied across clinicians. These factors collectively reduce the generalizability of the findings but reflect the complexity and heterogeneity of real-world clinical practice.

## Conclusions

ITC placement following ADP in obstetric patients was not associated with a statistically significant reduction in PDPH incidence or EBP requirement in our cohort. However, a trend toward reduced EBP use was observed, aligning with prior literature. Given the study's limitations, including retrospective design, small sample size, and procedural variability, our findings should be interpreted with caution. Rather than supporting a clinical recommendation, this study contributes region-specific, real-world data that reinforce the reproducibility of existing trends and highlight the need for standardized, prospective research. Future multicenter trials are essential to determine the optimal role, duration, and patient selection criteria for ITC use in obstetric anesthesia.
